# Reprogramming efficiency and quality of induced Pluripotent Stem Cells (iPSCs) generated from muscle-derived fibroblasts of mdx mice at different ages

**DOI:** 10.1371/currents.RRN1274

**Published:** 2011-10-27

**Authors:** Bo Wang, Yuko Miyagoe-Suzuki, Erica Yada, Naoki Ito, Takashi Nishiyama, Miho Nakamura, Yusuke Ono, Norio Motohashi, Makoto Segawa, Satoru Masuda, Shin'ichi Takeda

**Affiliations:** ^*^Department of Molecular Therapy, National Institute of Neuroscience, National Center of Neurology and Psychiatry, 4-1-1 Ogawa-higashi, Kodaira, Tokyo 187-8502, Japan; ^§^1.Department of Molecular Therapy, National Institute of Neuroscience, National Center of Neurology and Psychiatry, Kodaira 187-8502, Japan 2.Department of Biological Information, Tokyo Institute of Technology, Yokohama 226-8501, Japan and ^‡‡^1Department of Molecular Therapy, National Institute of Neuroscience, National Center of Neurology and Psychiatry, 4-1-1 Ogawa-higashi, Kodaira, Tokyo 187-8502, Japan 2Department of Life Sciences, The University of Tokyo, 3-8-1 Komaba, Tokyo 153-890, Japan

## Abstract

Induced pluripotent stem cells (iPSCs) hold promise as a potential treatment for Duchenne muscular dystrophy (DMD). To determine the impact of the donor’s age on reprogramming, we generated iPSCs from muscle-derived fibroblasts (MuFs) of mdx mice aged 6 weeks, 6 months, and 14 months. MuFs from 14-month-old mdx mice showed lower proliferative activity and lower reprogramming efficiency, compared with those from younger mdx mice. Furthermore, iPSCs derived from 14-month-old mdx mice (14m-MuF-iPSCs) gradually lost Nanog expression, and regressed in conventional ES medium during passages. Interestingly, inhibition of TGF-β signaling and BMP signaling stabilized Nanog expression and promoted self-renewal of 14m-MuF-iPSCs. Finally, rescued mdx-derived iPSCs efficiently differentiated into the skeletal muscle lineage.

## Introduction

Duchenne muscular dystrophy (DMD) is a severe muscle-wasting disorder caused by mutations in the dystrophin gene [1] [2]. There is currently no effective treatment for DMD, but stem cell therapy is expected to regenerate muscle tissues and improve muscle function. Induced pluripotent stem cells (iPSCs) are artificially reprogrammed to a state similar to that of embryonic stem cells (ESCs), i.e., capable of infinite growth and possessing pluripotency, by a defined set of transcription factors [3]. Because iPSCs can be induced from even severely affected DMD patients [4], patient-specific iPSCs are, in combination with genetic manipulation [5], expected to be a source of cells for autologous transplantation for treatment of DMD. 

Recent studies show that the nature of the original cell has profound impacts on both the efficiency of reprogramming and the properties of established iPSCs. Therefore, it is important to choose the optimal cell type for establishment of safe, high-quality iPSCs*.* Satellite cells/myoblasts might be good candidates [6], because it is proposed that epigenetic memories make iPS cells easily differentiate into the original cell type. But preparation of myoblasts is invasive. Skin fibroblasts are a more available source for clinical use. But, Miura et al. reported that adult mouse tail-tip fibroblasts-derived iPS cells tend to resist to differentiation induction and form tumors after transplantation [7]. Several groups recommend mature T-cells as a starting material, because blood sample is easy to obtain [8] [9] [10]. However, there are no conclusive data showing which cell type is the best for clinical use. In addition, recent studies showed that the reprogramming efficiency of aged cells is low [11] [12], but the mechanisms by which aged cells are refractory to reprogramming are not completely understood and the properties of aged cell-derived iPS cells remain to be evaluated. 

In this report, to analyze the impact of aging on the generation efficiency and properties of iPSCs, we derived iPSCs from muscle fibroblasts of *mdx *mice, a widely used model of DMD, at different ages. Muscle-derived fibroblasts (MuFs) from14-month-old *mdx* (14m-MuF-iPSCs) mice showed reduced replicative activity and lower reprogramming efficiency than those of younger *mdx *mice. Furthermore, iPSCs from 14-month-old *mdx* mice were unstable. Interestingly, inhibition of TGF-b and BMP signaling enabled us to obtain stable 14m-MuF-iPSCs. Finally, rescued 14m-MuF-iPSCs as efficiently differentiated into skeletal muscle lineage as iPSCs from younger mdx mice.

## Materials and Methods

### Animals 

C57BL/6-background *mdx* mice, which were generated by backcrossing of C57BL/6-background *mdx* to C57Bl/6 mice, were generously given by Dr. T. Sasaoka (Kitasato University School of Science, Kanagawa, Japan) were maintained in our animal facility. C57BL/6 and *NOD/Scid* mice were purchased from Nihon CLEA (Tokyo, Japan). All experimental procedures were approved by the Experimental Animal Care and Use Committee at the National Institute of Neuroscience (No. 2008014).

### Preparation of cells

Fibroblasts were isolated from whole hind limb muscles of 6-week-old (6w), 6-month-old (6m), or 14-month-old (14m) *mdx *mice. After removal of nerves, blood vessels, fat and connective tissues, muscle tissues were minced into small pieces by scissors, and plated onto culture dishes in MEF medium: Dulbeccos’ modified eagle medium (DMEM, Wako) containing 15% fetal bovine serum (FBS, Hyclone Laboratories), 1 mM sodium pyruvate (Invitrogen), 0.1 mM nonessential amino acids (NEAA) (Invitrogen), 2 mM L-glutamine (Sigma), 50 U/ml penicillin and 50 mg/ml streptomycin (Invitrogen). Mouse embryonic fibroblasts (MEFs) and tail-tip fibroblasts (TTFs) were prepared as described by Takahashi *et a* *l.* [13]. For reprogramming experiments, early-passage cells (<3 passage) were used.

### Retrovirus vectors

Plat-E packaging cells and pMX vectors were provided by Dr. T. Kitamura of the University of Tokyo [14]. The viral vector plasmids (pMXs-Sox2, pMXs-Oct4, pMXs-Klf4, and pMXs-c-Myc) were provided by Dr. S. Yamanaka of the University of Kyoto [3].pMXs-DsRed was generated by inserting DsRed cDNA (Clontech) into pMXs vector [15]. Plat-E cells were plated at 2x10^6^ cells per 60 mm collagen-coated dish (Iwaki). The next day, the cells were separately transfected with each retroviral vector plasmid by using FuGene 6 transfection reagent (Roche Diagnostics). Twenty four hours after transfection, the medium was replaced with new mouse embryonic fibroblasts (MEF) medium. After another 24 hours, the medium was collected, and concentrated to one-tenth using an Amicon Ultra centrifugal filter (Ultra-15) (Millipore).** ** 

### Generation and culture of iPSCs 

The concentrated virus-containing medium was added to fibroblast cultures. Two days later, the medium was changed to ES medium: DMEM (Wako) containing 15% FBS (Hyclone), 1 mM sodium pyruvate (Invitrogen), 0.1 mM NAA (Invitrogen), 2 mM L-glutamine (Sigma), 50 U/ml penicillin and 50 mg/ml streptomycin (Invitrogen), 0.11 mM 2-mercaptoethanol (2-ME) (Invitrogen), and 50 ng/ml recombinant mouse leukemia inhibitory factor (rLIF) (Millipore). Four days after retroviral transduction, fibroblasts were re-seeded on mitomycin C-treated MEFs at 1.0x10^4 ^cells/well in 6-well plates or 1.5-2.0x10^4 ^cells/dish in 60-mm dishes (IWAKI). The cells were maintained in a humidified atmosphere with 5% CO_2_ in air at 37°C. Two weeks after transduction, ES-like colonies were picked up and expanded. iPSCs were also maintained in the presence of valproic acid (VPA) (0.5 mM) (Sigma), Bix01294 (1 mM) (Stemgent), RG108 (1 mM) (Stemgent), PD0325901 (1 mM) (Stemgent), CHIR99021 (3 mM) (Stemgent), SB431542 (2.5 mg/ml) (Sigma), recombinant (r) BMP-7 (100 ng/ml) (R&D Systems), recombinant BMP-4 (100 ng/ml) (Stemgent), rNoggin (100 ng/ml)(R&D Systems), rTGF-β1 (100 ng/ml) (Stemgent), mouse rBMP-1a/ALK-3 Fc chimera protein (50 ng/ml), (R&D systems), human rBMPR-1b/ALK-6 Fc chimera protein (50 ng/ml) (R&D systems), or mouse sTGF-β RII Fc chimera protein (50 ng/ml) (R&D Systems).

### PCR analysis

Total RNA was extracted from cells using MicroRNeasy Kit (Qiagen). Complementary DNA was synthesized with QuantiTect reverse transcription kit (Qiagen). For primer sequences and conditions for RT-PCR analysis of *Eras, Fgf4, Oct4, Dax1, Nanog, Utf1, Cripto, Zfp296, Sox2, *and* Nat1*, we referred to Takahashi et al. [15]. *Pax7, MyoD, Myogenin,* *Tgfbr2, Acvr1, Acvr2a,* and *Bmpr2* were amplified using the following primers: *Pax7*, 5’-catccagtgctggtaccccacag-3’  and 5’-ctgtggatgtcacctgcttgaa-3’, *MyoD, *5’-aggctctgctgcgcgaccag-3’, and 5’-tgcagtcgatctctcaaagc-3’, *Myogenin,* 5’-tgagggagaagcgcaggctcaag-3’, and 5’-atgctgtccacgatggacgtaagg-3’ *Tgfbr2*, 5’-ggcttcactctggaagatgc-3’ and 5’-gggactgctggtggtgtatt-3’; *Acvr1*, 5’-cccaactctgaaacggacat-3’ and 5’- tgttgcatgggtaatggcta-3’; *Acvr2a*, 5’-gttacaccgaagccacccta-3’ and 5’-acaggagggtaggccatctt-3’; *Bmpr2*, 5’-ataggcgtgtgccaaaaata-3’ and 5’-attgtcaatggtgtgctgga-3’. PCR was performed on an iQ5 single color real-time PCR detection system (BIO-RAD). 

### FACS analysis and cell sorting

Cells were trypsinized, re-suspended at a concentration of 1.0x10^6^ cells / 100 ml in PBS containing 2% FBS, and incubated with CD31-PE (BD Bioscience), CD45-PE (BD Bioscience), Sca-1-PE(BD Bioscience), PDGFRa-PE (eBiosciences), or integrin a7-PE (MBL international corporation) antibodies. Analyses and cell sorting were performed on a FACSAria flow cytometer (BD Bioscience). 

### Cell staining

For AP activity, cells were stained with an Alkaline Phosphatase Substrate Kit III (Vector Laboratories, Inc.). The image was recorded with a microscope BIOREVO BZ-9000 (Keyence). For immunocytochemistry, cells were fixed with 4% paraformaldehyde for 5 min, permeabilized with 0.1% Triton-X in PBS for 10-30 min, blocked with 5% goat (Cedarlane) or horse (Invitrogen) serum in 2%BSA for 15 min, and then incubated with anti-Nanog rabbit polyclonal antibody (reproCELL), anti-sox2 antibody (6F1.2) (Millipore), anti-oct4 antibody (C-10) (Santa Cruz Biotechnology), mouse monoclonal beta III tubulin (2E9, Abcam), alpha-fetoprotein (AFP) (c-19) (Santa Cruz), mouse monoclonal cardiac troponin T antibody (1A11, Fitzgerald), mouse monoclonal anti-Pax7 antibody (PAX7, Santa Cruz), rabbit anti-MyoD antibody (Santa Cruz), mouse monoclonal anti-myogenin antibody (5FD, Santa Cruz) or anti-MHC antibody (MF20, R&D Systems). The specimens were then incubated with secondary antibodies labeled with Alexa-Flour 488 or 568 (Molecular Probes). Images were photographed using a fluorescence microscope IX71 (Olympus, Tokyo, Japan) equipped with Orca2 air-cooled CCD camera (Hamamatsu Photonics) and AQUACOSMOS software (Hamamatsu Photonics).

### In vitro differentiation

*In vitro* differentiation was performed as described by Chang and colleagues [16]. In brief, after culturing on gelatin-coated dishes without the feeder cells for three days, iPSCs were suspended in induction medium: high glucose DMEM (Wako) supplemented with 10% FBS (Tissue Culture Biologicals), 5% horse serum (Invitrogen), 0.1 mM NEAA (Invitrogen), and 0.1 mM 2-ME (Invitrogen), and were plated as hanging drops at a concentration of 800 cells/20 ml drop in 10 cm dishes. After 3 days culture, embryoid bodies were transferred to a suspension culture in 10 ml of induction medium in 10-cm low adherence dishes (Terumo) for 3 days, and then seeded on dishes coated with Matrigel basement membrane matrix (BD Biosciences, Bedford, MA, USA).  Myofibers appeared around the EBs in 30-60% of wells 7 days after plating EBs onto Matrigel-coated plates, and the fibers gradually grew and formed multinucleated fibers. 

### Teratoma formation 

iPSCs (1x10^6^) were subcutaneously injected into 6- to 8-week-old *NOD/Scid* mice. Five weeks after injection, tumors were dissected and fixed in 15% formalin, embedded in paraffin, cut by a microtome, and stained with hematoxylin and eosin (H. & E.).

### Genome-wide gene expression analysis 

Microarray analysis was performed by Toray Industries, Inc. (http://www.3d-gene.com). Total RNAs from iPSCs and ESCs (E14) were labeled with Cy3- or Cy5- using the Amino Allyl MessageAmp II aRNA Amplification Kit (Applied Biosystems). The Cy3- or Cy5-labeled aRNA pools were then hybridized with a 3D-Gene Mouse Oligo chip 24k in a buffer containing micro beads for 16 h. Data were analyzed by GeneSpring^TM^ software version 7.3.1 (Silicon Genetics).

### Statistical analysis

Results are expressed as mean+SD. Differences between groups were calculated for statistical significance using student’s *t-*test. p<0.05 was considered as significant.****

## **Results**

### Skeletal muscle-derived fibroblasts from 14-month-old *mdx* mice exhibited low proliferation activity, infection efficiency, and reprogramming efficiency 

To determine the impact of aging on the iPSCs generation, we isolated muscle fibroblasts (MuFs) from 6-week-old, 6-month-old, and 14-month-old *mdx* mice. FACS analysis showed that both WT-MuFs and mdx-MuFs are CD31(-)CD45(-)integrin a7(-)Sca-1(+) PDGFRa(+) (**Supplementary Figure 1**). In contrast, mouse embryonic fibroblasts (MEF) are heterogeneous in Sca1 expression, and negative for PDGFRa. Gene expression array analysis also showed that MEF and muscle fibroblasts are quite different in gene expression profiles (r^2^=0.67, data not shown). Muscle fibroblasts from 14-month-old *mdx *mice (14m-MuFs) proliferated more slowly (**Figure 1A**) than MuFs from younger *mdx *mice. Four reprogramming factors (*Sox2, Oct4(Pou5f1), Klf4, *and* c-Myc*) and* DsRed* were introduced by retroviral vectors into MuFs. The infection efficiency of 14m-MuFs (~30%) was much lower than those of 6w-MuFs (~60%), or 6m-MuFs (~60%). Alkaline phosphatase (AP) is an early marker of reprogramming. 14m-MuFs gave rise to fewer AP-positive colonies than those from younger *mdx *mice (data not shown). Next we sorted DsRed-positive cells by FACS and seeded them on feeders in 60 mm dishes, but the low reprogramming efficiency did not change (**Figure 1B**). Silencing of retrovirally-induced transgenes occurs at a later stage of reprogramming [17]. 14m-MuF- iPSCs exhibited significantly lower percentages of DsRed-negative colonies than those derived from younger mice (**Figure 1C, D**).

**Figure:**
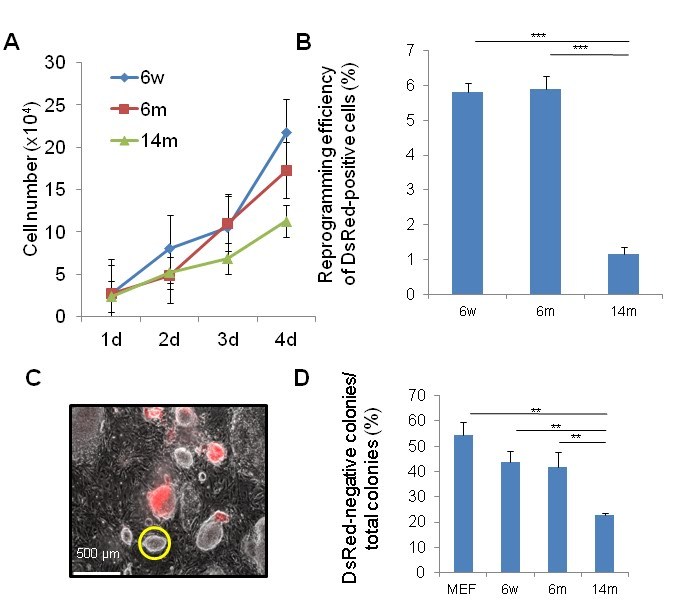
**Figure 1 **

**Muscle tissue-derived fibroblasts (MuFs) from 14-month-old *mdx* mice exhibit lower proliferation ability and reprogramming efficiency than those from younger mice **

(A)  MuFs from 6-week (6w), 6-month (6m) and 14-month-old (14m) *mdx* mice were seeded at 2.5x10^4^ cells/well on 12-well plates. Cell numbers were counted every day. Bar indicates mean + s.d. (n=4-6 mice/age group).

(B)   Four days after transduction, DsRed-positive cells were collected by a cell sorter and seeded at 1x10^4^/6-cm dish on MEF feeder cells. After two weeks, reprogramming efficiency (%) was calculated as in (C). Bar indicates mean + s.d. (n=3 mice /age group). ***p<0.001 (*t*-test)

(C)   Merged image of DsRed-positive and -negative colonies of 14m-MuF-iPSCs two weeks after transduction with retroviral vectors (*OKSM+DsRed*). DsRed-positive fibroblasts were collected and re-seeded on feeder cells. The transgenic DsRed gene is thought to be silenced in DsRed-negative colonies. The yellow circle indicates a typical ES-like DsRed-negative colony.

(D)  Percentages of DsRed-negative colonies after two weeks from retroviral transduction. Bar indicates mean + s.d. (n=3-6 mice/group). **p<0.01 (*t*-test).

### 14m-MuF-iPSCs are unstable, and regress with passaging 

To select fully reprogrammed iPSCs, we picked up DsRed-negative colonies with ES-like morphology. All clones expressed a battery of ES-specific genes (Supplementary **Figure 2A**). To further examine the properties of 14m-MuF-iPSCs, we picked up 50 DsRed-negative (retrovirus silenced) colonies derived from six 14-month-old *mdx* mice and expanded them *in vitro*. However, 14m-MuF-iPSCs clones were found to be unstable after 2-3 passages: many colonies regressed, and colony numbers dramatically decreased (**Figure 2B, C**). AP staining of 14m-MuF-iPSCs became weak or faint after expansion (**Figure 2B, C**). Similarly, Nanogexpression was gradually lost in 14m-MuF-iPSCs (**Figure 2B, C**). In contrast, nearly all 6w-iPSC and 6m-iPSC lines tested were stable and remained Nanog positive after further passages (**Figure 2B, C**). These results indicate that the four reprogramming factors are capable of inducing a pluripotent ES-like state in 14m-MuFs. However, in contrast to iPSCs from younger mice, the pluripotent state of 14m-MuF-iPSCs was unstable. 

To determine the molecular basis of the instability of 14m-MuF-iPSCs, three 14m-iPS clones derived from different mice were selected, and global gene expression analysis was performed. The Pearson correlation coefficient between 14m-MuF-iPSCs and ESCs was lower (r^2^=0.86-0.90) (n=3) than those between MEF-iPSCs and ESCs cells (r^2^=0.96) or 6m-iPSCs and ESCs (r^2^=0.95) **(Figure 4**). The expression levels of the genes involved in maturation and self-renewal of iPSCs (*Dppa4*,* Dppa3*,* Zfp42*,* Utf1*,* Nodal*,* Dnmt3I*,* Cldn3*,* Cldn4*,* Cldn7*,* Pou5t1*,* Lin28 *and* Sox2*) [18] in 14m-MuF-iPSCs were much lower than in ESCs, MEF-iPSCs or 6m-iPSCs (**data not shown**).

**Figure:**
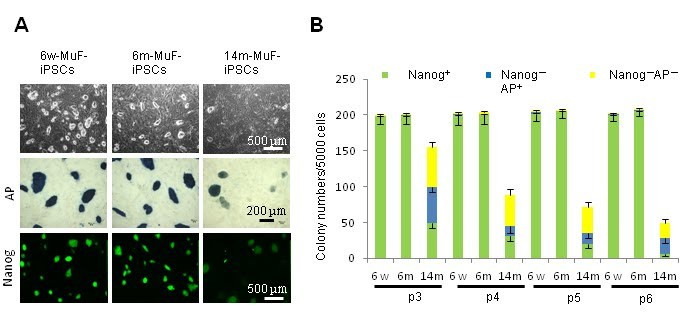
**Figure 2**

**iPSCs generated from MuFs of 14-month-old mdx mice are unstable, and regress during passaging**

(A) Representative phase images (PH), AP staining (AP), and immunofluorescence staining for Nanog of 6w-iPSCs, 6m-iPSCs, and 14m-MuF-iPSCs at passage 4.

(B) The number of Nanog- and AP-positive colonies (green), Nanog-negative and AP-positive colonies (blue), and Nanog- and AP-negative colonies (yellow) formed by 5000 cells were counted at passages 3, 4, 5 and 6. In contrast to iPSCs derived from younger mdx mice, both total and AP-positive and Nanog-positive colony numbers gradually decreased with passaging in 14m-MuF-iPSCs. (n=4-48 clones/age group).

### Inhibition of TGF-β and BMP signals endows 14m-MuF-iPSCs with self-renewal ability 

Interestingly, 14m-MuF-iPSCs exhibited higher mRNA levels of* BMPR2, TGF-βR2, Acvr2a, *and* Acvr1(ALK-2) *than other iPSCs or ESCs after 2-3 passages (**Figure 3A**), although there was no difference between the levels of these receptors in 14m-MuFs and younger MuFs (**data not shown**).To obtain stable 14m-MuF-iPSCs, we challenge several small molecules that were reported to improve reprogramming efficiency [19]. We found that TGF-β, BMP-4, or BMP-7 dramatically suppressed the propagation of 14m-MuF-iPSCs (**Figure 3B**), but not on ESCs or iPSCs generated from young mice (data not shown). We also added epigenetic modifiers to the culture medium of 14m-MuF-iPSCs. VPA, Bix01294, and RG108 did not increase the numbers of Nanog-positive colonies. A MEK inhibitor (PD0325901) and GSK3β inhibitor (CHIR99021), showed no beneficial effects on stabilization of 14m-MuF-iPSCs (**Figure 4C**). Interestingly, a TGF-β inhibitor, SB431542, which targets type I TGF-β receptors, Alk-4, Alk-5, and Alk-7 [20], and a BMP-antagonist, Noggin supported propagation of 14m-MuF-iPSCs, (**Figure 3C**). To confirm the effects of blockage of TGF-β or BMP signaling on 14m-MuF-iPSCs, we added soluble fragments of BMPR-1a (sBMPR-1a), BMPR-1b (sBMPR-1b), or TGF-β RII (sTGF-βRII) to the culture medium of 14m-MuF-iPSCs. Exposure to these soluble receptors resulted in an increase in the number of Nanog-positive colonies (**Figure 3D**). The treated cells formed ES-like colonies, grew rapidly, and continued to expressNanog (**Figure 3C**). Then, ES-like colonies were picked up again and cultured in ES medium without SB431542 and Noggin. Surprisingly, even after removal of the inhibitors, the number of Nanog positive colonies did not decrease during passages (**Figure 3E**).

**Figure:**
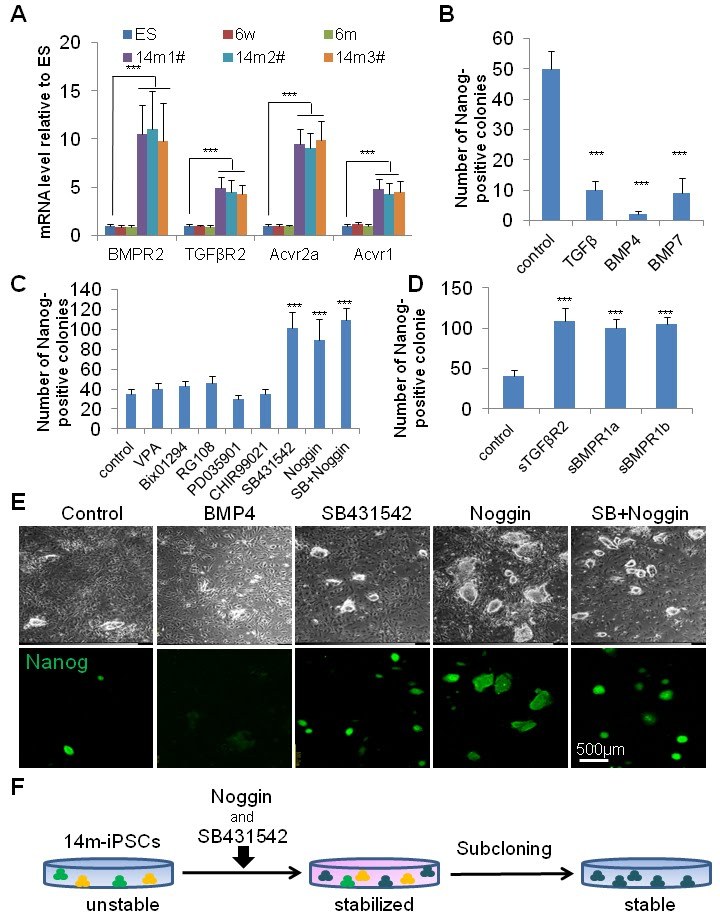
**Figure 3 **

**Inhibition of TGF-** **b** **and BMP signaling restores self-renewal of 14m-MuF-iPSCs**

 (A) qRT-PCR analysis for BMP-R2, TGF-βR2, Acvr2a, and Acvr1(ALK-2) of iPSCs derived from mice of different ages and ES (E14). All three 14m-iPS lines (#1, #2, and #3) showed higher expression levels of BMP-R2, TGF-βR2, Acvr2a, and Acvr1 mRNAs than iPSCs from younger mice and ES at passage 4 after being picked up. Similar data was obtained in three independent experiments. ***p<0.001 versus ES (t-test).

(B) Nanog-positive colonies in 14m-MuF-iPSCs (P4) was counted after treatment with TGF-β (100 ng/ml), BMP-4 (100 ng/ml), or BMP-7 (100 ng/ml). TGF-β and BMPs strongly inhibited colony formation of 14m-MuF-iPSCs. Data are presented as mean + s.d. (n=5 clones derived from different mice). ***p<0.001 versus control (t-test).

(C) 14m-MuF-iPSCs (P3) were plated onto MEF feeders and cultured in ES medium or addition of VPA (0.5 mM), Bix01294 (1 mM), RG108 (1 mM), PD035901 (1 mM), CHIR99021 (3 mM), SB431542 (2.5 mg/ml), or Noggin (100 ng/ml). SB431542 and/or Noggin significantly increased the number of Nanog-positive colonies. Bar indicates mean + s.d. (n=5 clones derived from different mice). ***p<0.001 versus control (t-test).

(D) 14m-MuF-iPSCs (P3) were plated onto MEF feeders and cultured in ES medium or ES medium supplemented with soluble TGF-βR2 (50 ng/ml), BMP-R1a (50 ng/ml), or BMP-R1b (50 ng/ml), and the number of Nanog-positive colonies were counted. (n=5 clones derived from different mice). ***p<0.001 versus control (t-test).

(E) Phase image and immunofluorescent staining for Nanog (green) of 14m-MuF-iPSCs in ES medium or treated with BMP-4, SB431542, Noggin, or a combination of SB431542 and Noggin at passage 4 after being picked up (P4).

(F) Subcloning of 14m-MuF-iPSCs after Noggin and SB431452 treatment. After OKSM-DsRed transduction of 14m-MuFs, DsRed-negative colonies were picked up and cultured in ES medium with SB431542 (2.5 mg/ml) and Noggin (100 ng/ml). After 2–3 passages, ES-like colonies were again picked up and expanded in ES medium without SB431542 and Noggin.

### Tail-tip fibroblasts (TTFs) from 14-month-old *mdx* mice show high proliferation potential, and give rise to stable iPSCs

To determine whether the low efficiency of reprogramming of fibroblasts from 14-month-old *mdx* mice is due to the aging, we isolated tail-tip fibroblasts from 14-month-old *mdx* mice (14m-TTFs). 14m-TTFs are CD31(-)CD45(-)integrin a7(-)Sca1(+) PDGFRa(+) (data not shown), and showed high viability (**Supplementary figure 2A**) and proliferation rate similar to mouse embryonic fibroblasts (MEFs). Importantly, 14m-TTFs showed much higher reprogramming efficiency (**Supplementary figure 2B**) than 14m-MuFs. AP staining and Nanog staining (**Supplementary figure 2C**), and global gene expression analysis (**Figure 4**) confirmed that iPSCs derived from 14m-TTFs were fully reprogrammed by four reprogramming factors. The established 14m-TTF-iPSCs were stable during passages (**data not shown**). These results suggest that the cellular senescence of 14m-MuFs, rather than the age of the animal, is a main cause for the low efficiency of reprogramming and the unstable state.

**Figure:**
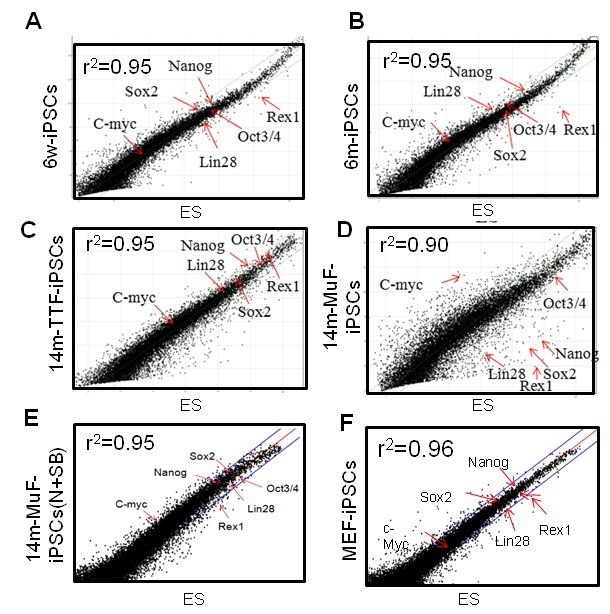
**Figure 4 **

**14m-MuF-iPSCs show different gene expression pattern from ESCs, but SB431542 and Noggin-treated 14m-MuF-iPSCs show ES-like expression profiles**

Scatter plots comparing global gene expression profiles of 6w-iPSCs (A), 6m-iPSCs (B), 14m-TTF-iPSCs (C), 14m-MuF-iPSCs (p3) (D), inhibitor-treated 14m-MuF-iPSCs (E), and MEF-iPSCs (F) to ESCs (E14). SB+N: SB431542 and/or Noggin.

### Characterization of 6w-, 6m-, 14m-MuFs-, and 14m-TTFs-iPSCs

After SB431642 and Noggin treatment, three sub-lines of 14m-MuF-iPSCs were established (**Supplementary figure 3**), and maintained in ES medium. To determine whether iPSCs derived from mdx mice were indeed pluripotent, we assessed the expression of pluripotent markers. All mdx-derived iPSCs were positive for Sox2, Oct4, and Nanog (**Supplementary figure 3B**). Next, we evaluated the in vitro differentiation capacity of iPSCs by formation of embryoid bodies (EBs) in suspension culture. All iPSCs tested formed EBs after 6 days of culture and differentiated into different cell types as detected by the expression of neuron-specific tubulin (Tuj1), toroponin T (cardiac), and endodermal α-fetoprotein (AFP) after attachment on Matrigel-coated plates (**Supplementary figure 3C**). Lastly, we tested the ability of these cells to generate teratomas in *NOD/Scid* mice. Six to eight weeks after injection, all iPSCs formed teratomas containing complex tissue structures characteristic of all three germ layers (**Supplementary figure 3D**). 

### All 6w-MuF-, 6m-MuF-, 14m-MuF-, and 14m-TTF-iPSCs efficiently differentiate into skeletal muscle lineage *in vitro*

To evaluated myogenic potential of mdx-derived iPSCs, we applied a directed skeletal muscle differentiation protocol as described in M&M (**Figure 5A**) [16] [21]. RT-PCR revealed Pax7, MyoD and myogenin expression as early as day13 (**Figure 5B**). All *mdx*-derived iPSCs formed EBs and differentiated into multinucleated muscle fibers (**Figure 5C**). Immunocytochemistry confirmed the expression of Pax7, MyoD, myogenin, and myosin heavy chain (MHC) (**Figure 5C,** **Supplementary Figure 4**). In this culture system, we found numerous Pax7-positive cells and Pax7-positive/MyoD-positive cells (**Figure 5D**) two weeks after plating EBs on Matrigel plates, indicating that myogenic stem/progenitor cells were successfully induced in all *mdx*-derived iPSCs. 

**Figure:**
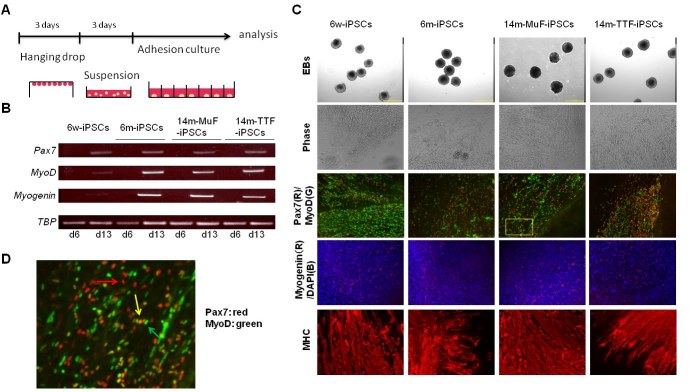
**Figure 5 **

**Differentiation of iPSCs derived from mdx of different ages into skeletal muscle lineage *in vitro*.**

(A) Schematic representation of our myogenic differentiation system.

(B) The expression of myogenic genes during iPSCs differentiation examined by RT-PCR.

(C) Phase images showing embryoid bodies (EBs) (day 6) and myotubes (day 21), and immunocytostaining of differentiated cells derived from 6w-, 6m-, and 14m-MuFs and 14m-TTFs-iPSCs, for Pax7(red) and MyoD(green), myogenin(red), and MHCs(red). Nuclei were stained with DAPI.

(D) High-power magnification of a marked region in C, showing Pax7 single-positive (red arrow), MyoD-single positive (green arrow) or double- positive cells (yellow arrow).

## **Discussion**

In this study, we examined the reprogramming efficiency and quality of iPSCs generated from muscle-derived fibroblasts (MuFs) of *mdx* mice at different ages, and found that muscle fibroblasts (MuFs) from 14-month-old *mdx *mice showed lower proliferative activity and lower reprogramming efficiency, compared with those from younger *mdx* mice. In addition, 14m-MuF-iPSCs gradually lost Nanogexpression, and regressed in conventional ES medium during passages. In contrast, tail-tip fibroblasts prepared from 14-month-old *mdx *mice were highly proliferative, and efficiently reprogrammed, and stable in culture. These observations suggest that poor reprogramming can not be simply attributed to the age of the mice. Dystrophin deficiency causes continuous degeneration/regeneration of skeletal muscle, and older mdx muscles show fibrosis (**Supplementary Figure 1**). Interestingly, FACS analysis showed that muscle fibroblasts are CD31(-)CD45(-)integrina7(-)Sca1(+)PDGFRa(+), suggesting that they are muscle-resident fibro/adipogenic progenitors (FAPs), which were recently demonstrated to proliferate in response to damage and facilitate myogenesis but differentiate into adipocytes and promote fibrosis in pathological conditions [22] [23]. Therefore, the low reprogramming efficiency of 14-month-old mdx fibroblasts might be largely explained by the exhaustion of fibroblasts due to repeated activation. To further clarify aging effects on reprogramming of muscle fibroblasts, we are now comparing 14-month-old mdx muscle-derived fibroblasts side-by-side with 14-month-old WT muscle fibroblasts. 

Several chemicals have recently been reported to either enhance reprogramming efficiencies or substitute for specific reprogramming factors [19], including chromatin modifiers, DNA methyltransferase inhibitors [24] or TGF-β pathway inhibitors [25] [26]. In this study, we tested these chemicals to rescue unstable 14m-MuF-iPSCs, and found that inhibition of TGF-β signaling or BMP signaling can rescue the unstable iPSCs. By using SB431542 and noggin, we successfully subcloned several stable iPSCs from parental 14m-MuF-iPSCs. Maherali and Hochedlinger reported that TGF-β inhibition is most effective at the initial stage of reprogramming [20]. Li and colleagues demonstrated that inhibition of TGF-β signaling accelerates the reprogramming process by suppressing pro-EMT signals and activating an epithelial program [18]. In contrast, Ichida et al. showed that a TGF-β inhibitor can replace Sox2 byacting on cellular intermediates at later time points [26]. Our results, however, suggest that TGF-β signaling is also involved in destabilization of iPSCs in the final stage of reprogramming. In this report, we demonstrated that Noggin, soluble BMPR-1a, or soluble BMPR-1b supported the expression of Nanog and the growth of 14m-MuF-iPSCs. In contrast, BMP-7 and BMP-4 further destabilized 14m-MuF-iPSCs. Importantly, these effects were not observed in iPSCs from younger mice (**data not shown**). Previous studies showed that BMPs support self-renewal of mouse ESCs [27] [28]. Recent studies further showed that BMPs promote reprogramming at an early phase by initiating the MET transition [18]. The gene expression pattern suggests that at an early passage, 14m-MuF-iPSCs are not fully reprogrammed, probably between the maturation and stabilization stages. Further analysis on the mechanisms by which noggin promotes self-renewal of 14m-MuF- iPSCs is needed. 

Importantly, we found mdx-iPSCs efficiently differentiate into myofibers. In the culture, we found numerous Pax7-positive cells and Pax7-positive/MyoD-positive cells in the muscle induction system. This observation is promising, because Pax7 is a marker of muscle stem/ progenitor cells and muscle satellite cells [29]. In combination with *ex vivo* gene transfer technique, patient-derived iPS cells are a promising tool to combat degenerative muscle disorders.

##  Acknowledgements 

We thank all members of the Department of Molecular Therapy for technical support and discussion. We also appreciate Ryoko Nakagawa for technical assistance.

## Funding information

This work was supported by a research grant for Neuromuscular Diseases (19A-7), a research grant for Nervous and Mental Disorders (20B-1), Health and Labor Sciences Research Grants for Translation Research (H19-Translational Research-003 and H21-Clinical Research-015), Health Sciences Research Grants for Research on Psychiatry and Neurological Disease and Mental Health (H18-kokoro-019) from the Ministry of Health, Labour and Welfare, Grants-in-Aid for Scientific Research (18590392; 20590418) and a grant for the realization of regenerative medicine from the Ministry of Education, Culture, Sports, Science and Technology.

## Competing interests 

The authors indicate no potential conflicts of interest.

**Figure:**
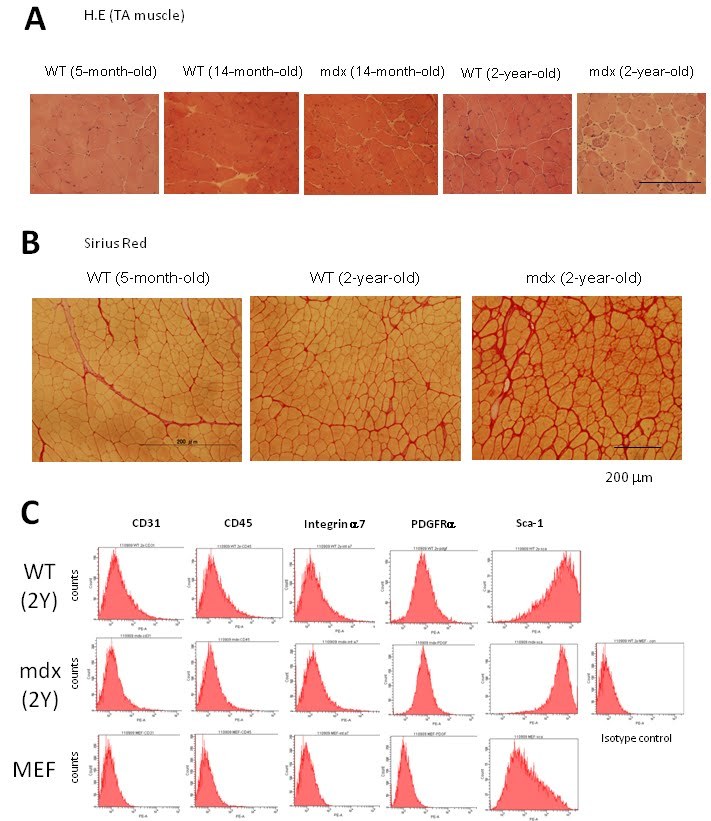
**Supplementary figure 1**

**Fibrotic changes in* mdx* muscle and characterization of muscle-derived fibroblasts.**

(A)  H.E staining of wild-type and dystrophic mdx TA muscle at different ages. Note increased interstitial tissues in mdx muscle with age.

(B)  Sirius Red staining of WT and mdx TA muscle. TA muscle of 2-year-old wild type muscle also shows increased interstitial tissue.

(C)  FACS analysis for CD31, CD45, integrin a7, PDGFRa, and Sca1, of muscle fibroblasts prepared from 2-year-old mdx muscle.

**Figure:**
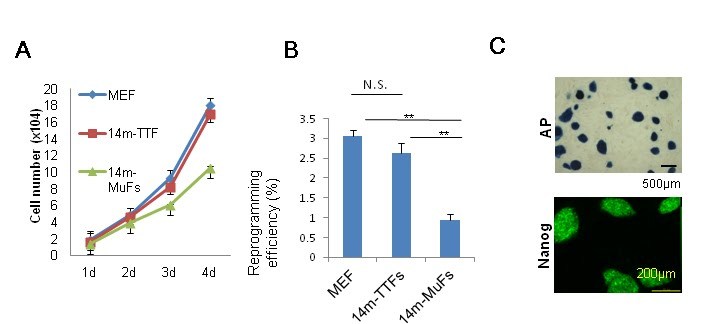
**Supplementary Figure 2 **

**Tail-tip fibroblasts (TTFs) from 14-month-old *mdx* mice show high proliferation potential, and give rise to stable iPSCs **

(A) 14m-TTFs and MEFs exhibit similar proliferation ability. MEFs, 14m-TTFs, and 14m-MuFs were seeded at 2.5x10^4 ^cells /well on 12-well plates. Cell numbers were counted every day. Bar indicates mean + s.d. (n=3/group).(B) 14m-TTFs and MEFs show similar reprogramming efficiency. Four days after infection of a cocktail of retrovirus vectors (OKSM+DsRed), DsRed-positive cells were re-seeded at 1.35x10^4^ cells/6-cm dish on feeder cells. Ten days later, colonies were stained for alkaline phosphatase (AP). Reprogramming efficiencies are shown as AP+ colonies per seeded cells. Bar indicates mean + s.d. (n=3/group). **p<0.01 (t-test).

(C) Representative AP and Nanog staining of 14m-TTF-iPSCs.

**Figure:**
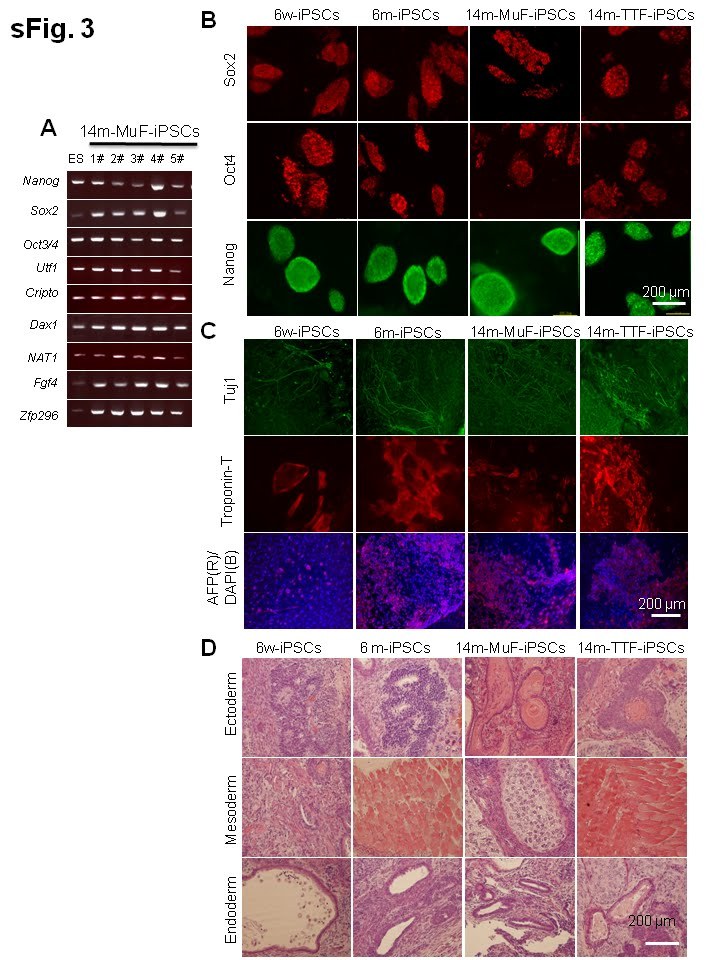
**Supplementary figure 3**

**ES-like properties of iPSCs established from *mdx *mice of different ages**

(A)    RT-PCR analysis of pluripotent markers in five lines of 14m-MuF-iPSCs derived from different 14-month-old mdx mice.

(B)     Immunofluorescent staining for Sox2, Oct4, and Nanog of 6w-iPSCs, 6m-iPSCs,14m-MuF-iPSCs, and 14m-TTF-iPSCs.

(C)     iPSCs of different origins differentiate into bIII tubulin-positive ectodermal cells (green) (upper panel), cardiocytes (troponin T, red) (middle panel) and alpha-fetoprotein (AFP)-positive endodermal cells (red) (lower panel) in vitro. After formation of embryoid bodies, cells were plated on Matrigel-coated plates. Ten days later, the cells were fixed and stained with specific antibodies.

(D)    Well differentiated teratomas formed by 6w-iPSCs, 6m-iPSCs, 14m-MuF-iPSCs, and 14m-TTF-iPSCs.

**Figure:**
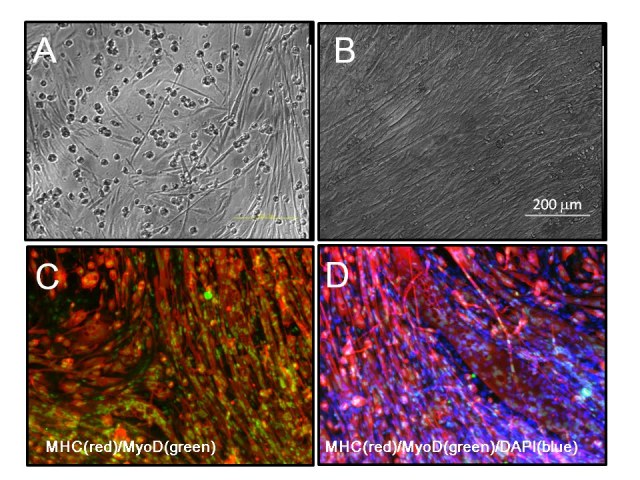
**Supplementary Fig.4**

**Muscle differentiation of 14m-MuF-iPSCs **

(A and B) Phase images of 14m-MuFs-iPSCs-derived myofibers.

(C) MHC(red) and MyoD (green) staining of 14m-MuFs-iPSCs-derived myofibers.

(D) Merged image of MHC(red), MyoD(green), and DAPI(blue) staining of 14m-MuFs-iPSCs-derived myofibers.
